# EVI1 expression in childhood acute lymphoblastic leukaemia is not restricted to MLL and BCR/ABL rearrangements and is influenced by age

**DOI:** 10.1038/bcj.2013.76

**Published:** 2014-01-24

**Authors:** A Stevens, D Hanson, C de Leonibus, A Whatmore, R Donn, D J White, J Liu, M M van den Heuvel-Eibrink, V Saha, P E Clayton, S Meyer

**Affiliations:** 1 Department of Paediatric Endocrinology, Royal Manchester Children's Hospital, Manchester, UK; 2 Manchester Academic Health Science Centre, University of Manchester, Royal Manchester Children's Hospital, Manchester, UK; 3 The Centre for Musculoskeletal Research, Manchester, UK; 4 Stem Cell and Leukaemia Proteomics Laboratory, Manchester, UK; 5 Children's Cancer Group, Manchester, UK; 6 Institute for Cancer Sciences, University of Manchester, Manchester, UK; 7 Department of Pediatric Hematology and Oncology, Erasmus MC-Sophia Children's Hospital, Rotterdam, The Netherlands; 8 Tata Translational Cancer Research Centre, Kolkata, India; 9 Department of Paediatric Oncology, Royal Manchester Children's Hospital, Manchester, UK; 10 Young Oncology Unit, The Christie NHS foundation Trust, Manchester, UK

EVI1 is a transcriptional regulator with an important function in haematopoiesis and self-renewal.^1^ Aberrant overexpression of *EVI1* has been firmly established as one of the most adverse prognostic markers in acute myeloid leukaemia (AML),^2^ implying that *EVI1* is one of the most aggressive oncogenes in AML. Importantly, a recent report in *Leukemia* from Konantz *et al.*^3^ suggests that *EVI1* might also have a role in paediatric acute lymphoblastic leukaemia (ALL), where high expression confers apoptosis resistance, and possibly also an adverse prognosis. Rearrangements of the 3q26 region, which encompasses the *MECOM* (MDS–EVI1 complex) gene that encodes *EVI1* transcripts and that are commonly associated with *EVI1* overexpression in adult AML, rarely occur in childhood ALL or AML.^2, 4^ However, when *EVI1* is expressed in childhood AML it seems to be predominate in *MLL*-rearranged cases,^4, 5^ which may then confer an adverse prognosis, as illustrated by the correlation with the t(6;11) subtype,^4^ and with complex karyotype cases. However, in general high *EVI1* expression is not seen in AML with good prognosis cytogenetics such as core-binding factor-rearranged AML with t(8;21) or inv(16)^4, 5^ (Figure 1a). In addition, in chronic myeloid leukaemia, the BCR–ABL fusion tyrosine kinase sustains *EVI1* expression.^6^

To complement the work reported by Konantz *et al.*,^3^ we analysed gene expression data generated from nucleated cells obtained from diagnostic bone marrow aspirates of 70 *de novo* ALL (31 female subjects, median age at diagnosis: 4.4 years, range: 1.1–14.6 years), using the Affymetrix U133+2 platform from our previously published and verified data set.^7, 8^ We analysed nine T-cell ALL, three BCR–ABL-rearranged Philadelphia-positive, one MLL-rearranged and 57 ALL with other cytogenetics, of which most were hyperdiploid. We focussed on *EVI1* transcripts targeted by probes 226420_at and 221884_at, which detect exonic sequences of *EVI1* transcripts.^5^ We also included two large and sufficiently annotated gene expression data sets in our analysis, together comprising 455 *de novo* ALL samples (GEO Data sets GES28497^9^ and GES13425^10^). To determine whether high *EVI1* expression in ALL is also associated with specific chromosomal changes involving *BCR/ABL* or *MLL* rearrangements, or T-cell phenotype, we divided *de novo* childhood ALL into four subgroups. We analysed T-cell ALL separately from *BCR–ABL* fusion (Philadelphia)-positive, *MLL* gene rearrangement-positive and ALL with other cytogenetic abnormalities (the largest group). In addition, and separately, we also analysed paired data sets of *de novo* and subsequently relapsed childhood ALL (GSE28460).^11^

*EVI1* expression is variable in *de novo* paediatric ALL. However, the range of *EVI1* expression levels is much smaller as compared with childhood AML (Figure 1a). When applying the criteria used for paediatric AML with samples considered *EVI1*-positive (+) when *EVI1* expression is higher than log0.5 normalised fold change, the proportion of *EVI1*+ ALLs is larger than AML and includes cases of T-cell ALL. There was no clear association of higher *EVI1* expression levels with *BCR–ABL* or *MLL* rearrangements in the analysis of our own samples and results of published data sets (Figure 1a). The variability of *EVI1* expression in the *MLL*-rearranged ALL group might be partly dependent on specific *MLL* gene rearrangements, as AMLs with *MLL/AF6* and *MLL/AF9* fusions are associated in particular with high *EVI1* expression in adults and children.^5, 12^ We found no obvious possible cause for variability of *EVI1* expression in ALL of other cytogenetic subgroups. Given the rarity of 3q rearrangements in ALL, high *EVI1* expression is likely to be a secondary event. Cytogenetic features of high *EVI1*-expressing ALL, which include hyperdiploid and normal karyotype disease, are listed in Supplementary Table 1.

Konatz *et al.*^3^ observed that *EVI1* modulated the expression of apoptosis-related genes in paediatric ALL. In accordance with their data,^3^ we identified *BCLX* (fold change −1.8, *P*=0.007) and *PUMA* (FC=−1.9, *P*=0.005) in T-cell ALL when determining *EVI1*-co-regulated genes by selecting for significantly changed (*P*<0.01, analysis of variance) low and high expressed genes in paediatric T- and B-cell ALL with *EVI1* expression >log1.5. To determine the overlap in global expression patterns associated with high *EVI1* transcripts, we chose a >log1.5 cutoff to analyse a meaningful sample size for comparison. In general, the overlap was greater between T-cell ALL and B-cell ALL than either of these groups with AML (Supplementary Information, Figure 1). Only 12 genes were significantly co-regulated in all the subgroups of childhood leukaemia (*P*<0.01, analysis of variance) (Supplementary Information, Figure 1), with seven regulated in different directions between the subgroups. This included *SMARCA5*, of which the encoded protein recently has been shown to directly interact with the EVI1 protein in SKOV ovarian cancer cells and K562 leukaemia cells.^13^

Importantly, we noted that several of the high *EVI1*-expressing ALLs were from patients in late childhood or adolescence. To further explore a possible association of *EVI1* expression with age in *de novo* childhood ALL, we carried out rank regression analysis by age on our B-cell ALL cases, which is the largest group of our age- and sex-annotated data set (*n*=51), excluding those with *MLL* or *BCR–ABL* rearrangements. We found a highly significant increase in *EVI1* expression with age at diagnosis of ALL (Figure 1bi, upper panel) during childhood years (age 1–10, *r*=0.29, *P*=0.05) but in particular obvious with the onset of adolescence (age 1–14, *r*=0.53, *P*<0.003). To further explore the relationship of patient age on gene expression patterns in childhood ALL we applied this approach to the entire data set. We identified 415 probe sets corresponding to 341 genes with significant age-associated changes in expression levels (*P*=0.01) (Figure 1bii, lower panel). Of these, 130 genes have the highest expression in adolescence (cluster 3). When we associated the upstream regulators of these 130 genes (Ingenuity pathway analysis), we found that 21 are regulated directly by transforming growth factor-β (*P*=1.8 × 10^−3^). This resembles our findings with respect to the impact of age on gene expression patterns in general^14^ and implies that transforming growth factor-β might in particular contribute to age-dependent changes in ALL gene expression observed in the adolescent age group. As *EVI1* also has been shown to have an impact on transforming growth factor-β signalling,^15^ we have started to investigate this in more detail. When we analysed the age association of gene expression patterns in our AML data set (also excluding *MLL*-rearranged cases), we detected some striking differences to ALL. Although age has an impact on gene expression patterns also distinctly in AML, we see a negative correlation of *EVI1* with increasing age (Supplementary Figure 2).

The data set by Hogan *et al.*^11^ shows that in *de novo* ALL that subsequently relapses *EVI1* expression has a significantly wider range compared with the *de novo* ALL cases that are not selected for subsequent relapse in the other studies (*P*<0.01). Importantly, paired analysis suggests that at ALL relapse, expression of *EVI1* is on average higher than in the corresponding *de novo* sample (1.3-fold increased, *P*<0.006, Figure 1c).

In summary, our analysis confirms high *EVI1* expression in a group of paediatric ALL, which does not appear to be confined to distinct cytogenetic subtypes. The limited overlap of gene expression profiles associated with high *EVI1* expression in different forms of childhood leukaemia and the different levels of expression of some co-regulated genes implies the possibility of tissue specificity, and the impact of cell of origin for *EVI1*-mediated transcriptional regulation, which has also been suggested for *MLL*-rearranged AML subtypes.^16^ High *EVI1* expression itself is likely to be a secondary event in paediatric lymphoblastic and myeloid leukaemia. The higher expression in older children might be linked to the worse prognosis of ALL in this age group. Whereas higher *EVI1* expression in relapsed disease might be a function of increasing age, it also implies potentially a more general role in ALL stem cell survival. It will be important to prospectively investigate *EVI1* in paediatric ALL for prognosis on current treatment regimens, and potential therapeutic benefit. In addition, our analysis strongly suggests an impact of age at diagnosis on gene expression patterns in childhood leukaemia. As we are uncertain to what extent this reflects the chronological age of the patient, or that of the cell of origin in childhood leukaemia, we have started to investigate this more comprehensively.

## Figures and Tables

**Figure 1:**
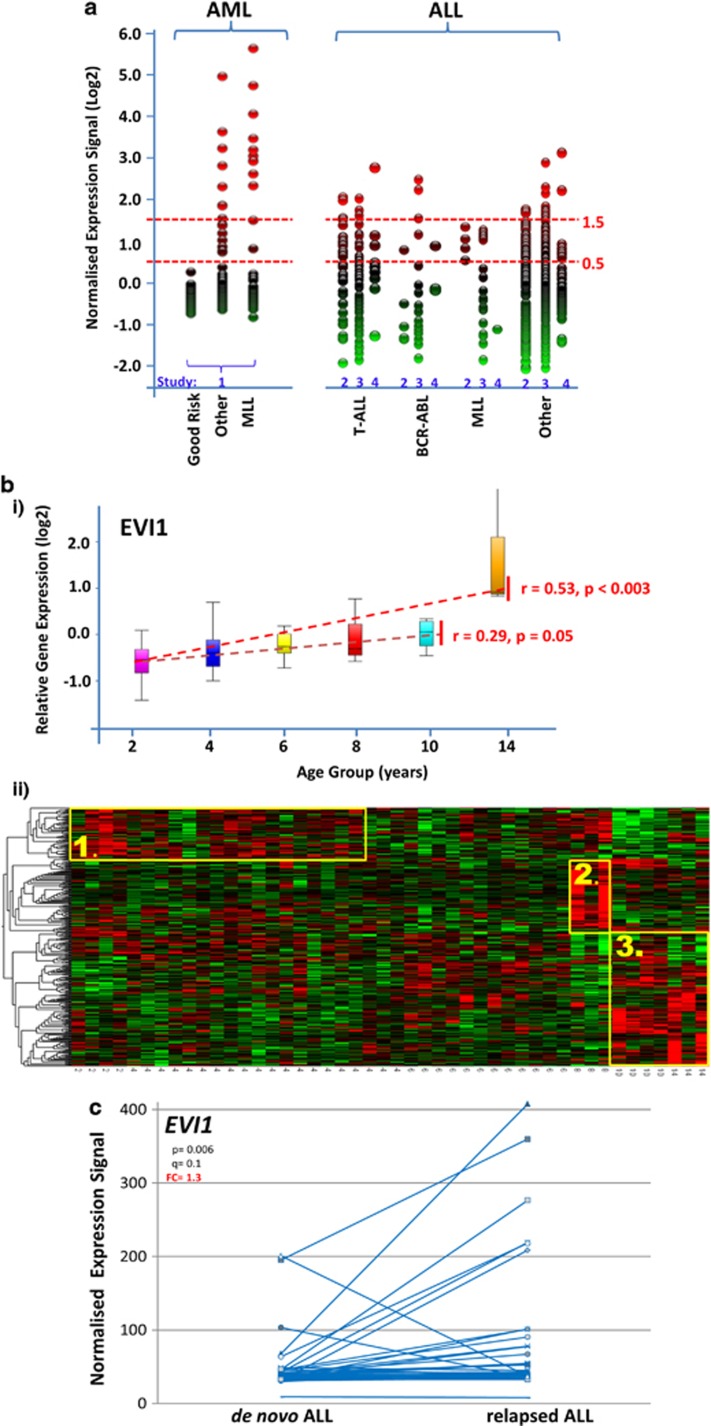
(**a**) Variability of *EVI1* expression in paediatric leukaemia. Comparison of *EVI1* expression of using the *221884_at* Affymetrix gene probe. In an AML data set (1 GSE17855, *n*=237), Good Risk=good risk cytogenetics inv(16), t(15;17) and t(8;21) (*n*=74), Other=remaining cytogenetics (*n*=116) and MLL=*MLL* gene rearrangement-positive (*n*=47). The comparison shows three data sets of ALL (two GSE 13425 (*n*=190), three GSE28497 (*n*=255) and four Manchester (*n*=62)); BCR–ABL=BCR–ABL fusion (Philadelphia)-positive (*n*=5, *n*=16, *n*=3); T-ALL=T-cell ALL (*n*=36, *n*=45, *n*=9); MLL=*MLL* gene rearrangement-positive (*n*=4, *n*=18, *n*=1), Other=other ALL (*n*=145, *n*=176, *n*=49). (**b**) Age-associated gene expression in ALL panel (I). Age-related expression of *EVI1* as measured by *221884_at* Affymetrix gene expression probe set in 46 individuals with ALL (4.1, 1.1–13.0 years of ages (median, range); 23 female individuals); T-ALL, BCR–ABL and MLL-rearranged groups were removed. Box plot by age group (upper limit of bin shown), dotted line=median, whiskers show top and bottom quartiles. Rank regression by age, *P*-value and *r*-value shown. Panel (II) Heat map of age-associated changes in gene expression from cells obtained from diagnostic bone marrow aspirates of 46 individuals with ALL. Gene probe sets associated with age by multigroup analysis of variance with gender as covariate, *P*<0.01, identifying 415 probe sets as age related corresponding to 341 unique genes. Unsupervised hierarchical clustering using Euclidean metric with each variable normalised to mean 0 and variance 1. The horizontal axis ranked by age group (in years), age-related clusters derived from the dendrogram (vertical axis) highlighted in yellow; cluster 1=infancy/early childhood, cluster 2=late childhood, cluster 3=late childhood/puberty. Age specificity of clusters was confirmed using random substitution of probe sets (Qlucore Omics Explorer 2.3). (**c**) *EVI1* expression in paired samples of *de novo* and relapse of ALL. Comparison of gene expression at diagnosis and at relapse of ALL from GSE28460 (Hogan *et al.*^11^). Expression of *EVI1* was measured using the *221884_at* Affymetrix gene probe set. Affymetrix gene expression microarray data normalisation was confirmed using multidimensional scaling in Qlucore Omics Explorer 2.3 and analysis of gene expression change was undertaken using a paired *t*-test with gender as an eliminated factor; q=false discovery rate-modified *P*-value, FC=fold change.
