# Supplementary material for: EVI1 expression in childhood acute lymphoblastic leukaemia is not restricted to MLL and BCR/ABL rearrangements and is influenced by age
Source: Blood Cancer J. 2014 Jan 24;4(1):e179–. doi: 10.1038/bcj.2013.76 (PMC3913945; doi:10.1038/bcj.2013.76)
Supplement: Supplementary Information [file bcj201376x1.doc]

**Supplementary information Stevens *et al***

***EVI1 expression in childhood acute lymphoblastic leukaemia is not restricted to MLL and BCR/ABL rearrangements and is influenced by age***

**Supplementary Table 1.**

**Title: Cytogenetic changes in samples with high *EVI1* Expression.**

**Legend:** Cytogenetic changes in the Manchester data set along with age and gender for samples with high gene expression of *EVI1* (defined as *EVI1* > 0.5 log2 normalised gene expression signal as measured by Affymetrix probe set *221884_at*) [n =12/49].

**Supplementary table 1**

| **Sample** | **Cytogenetics** | **Gender** | **Age at diagnosis** |
| --- | --- | --- | --- |
| 1 | Failed | F | 12.95 |
| 2 | 57, XX, +X, +X, +der(1)t(1;?),+4,+6,+10,+14,+17,+18,+21,+21[7]/46,XX[3]  (triple-test negative) | F | 5.25 |
| 3 | 59,XX,+X,+2,+4,+6,+8,+9,+10,+12,+17,+?18,+21,+21,+21,inc(6) | F | 8.58 |
| 4 | 45,XX,der(7;9)(q10;q10) | F | 3.17 |
| 5 | 46,XX, del(6)(q1q2), i(8)(q10), ?add(21)(q22);48, XX der (8;9)(q10;q10), ?add(12)(p1); add(21)(q22), +add(21)(q22),+mar (TEL-AML1) | F | 12.17 |
| 6 | 46,XY | M | 3.75 |
| 7 | t(12;21) and TEL loss in 73% | M | 5.5 |
| 8 | 46,XY,dup(7)(p15p22),t(12;19)(q13;p13)(20), TELAML1(+) in 100% interphase cells,  additional copies of AML1 in 36%of interphase cells | M | 9.17 |
| 9 | 55, XX, +X, +4, +6, +8, +10. +14, +18, +19 ?del(20)(q1?), +21 (9) | F | 3.83 |
| 10 | 46,XY, i(17)(q10)(2)/46,XY,idem,?dup(7)(p?21),del(13)(q?14,q?22)(1)/46,XY,idem,?dup(7)(p?21)  ,del(13)(q?14q?22),+mar1,+mar2(1),/46,XY(9) | M | 12.38 |
| 11 | 57,XY,+X,+4,+5,+9,+10,+14,+17,+?add(18),(q?11.2),+21c,+mar(1)/58,idem,+21 (5)/59,idem,+11,+21(2)/47,XY,+21c (4) | M | 3.75 |
| 12 | complex | M | 7.75 |

**Supplementary Figure 1**

**Title: Gene expression changes associated with high *EVI1* expression*.***

**Legend:** Gene expression data sets were curated to compare samples with high gene expression of *EVI1* [as measured by Affymetrix probe set *221884_at*]; patients with high expression of *EVI1* (defined as ≥ 1.5 log2 normalised gene expression signal) were compared to the remaining patients (ANOVA, p<0.01). For the AML dataset (GSE17855) expression of *EVI1* ≥ 1.5 log2 [n=18/237] was associated with 2079 probe-sets equating to 1299 separate genes. For the T-Cell ALL data (union of GSE13425 & GSE28497) expression of *EVI1* ≥ 1.5 log2 [n=4/36 & 3/45] was associated with 644 probe-sets equating to 595 separate genes. For the group “Other ALL” (union of GSE13425, GSE28497 and Manchester data) expression of *EVI1* ≥ 1.5 log2 [n=3/145 & 15/176 & 2/49] was associated with 2283 probe-sets equating to 1946 separate genes. The 12 genes that are associated with high *EVI1* expression in all groups are shown with condition related expression marked (red = increased expression, grey = decreased expression). Venn diagrams are of gene numbers.

**Supplementary Figure 1:**

**Supplementary Figure 2**

**Title: 2. Age-associated gene expression in childhood AML**

**Legend:**

**Panel I)** Age-related gene expression of EVI-1 as measured by *221884_at* Affymetrix gene expression probe set in 76 individuals with AML (8.0, 2.0-14.0 years of ages [median, range]; 36 female & 40 male), “good risk” and MLL groups were removed. Box plot by age group (upper limit of bin shown), dotted line = median, whiskers show top and bottom quartiles. Rank regression by age, p-value and r-value shown.

**Panel II)** Heat map of age-associated changes in gene expression from peripheral blood mononuclear cells in 76 individuals with AML. Gene probe sets associated with age by multi-group ANOVA with gender as co-variate, p<0.01, identifying 2045 probe-sets as age related corresponding to 1411 unique genes. Unsupervised hierarchical clustering using Euclidean metric with each variable normalised to mean 0 and variance 1. Horizontal axis ranked by age group (in years), age-related clusters derived from the dendrogram (vertical axis) highlighted in yellow; cluster 1 = infancy/early childhood, cluster 2 = mid-childhood, cluster 3 = early childhood, cluster 4. = late childhood/puberty. Age specificity of clusters was confirmed using random substitution of probe-sets (Qlucore Omics Explorer 2.3).
